# Supplementary material for: Growth, physiological and transcriptomic analysis of the perennial ryegrass Lolium perenne in response to saline stress
Source: R Soc Open Sci. 2020 Jul 8;7(7):200637. doi: 10.1098/rsos.200637 (PMC7428229; doi:10.1098/rsos.200637)
Supplement: supplementary materials.docx [file rsos200637supp1.docx]

**Supplementary information**

**Growth, physiological and transcriptomic analysis of the perennial** **ryegrass *Lolium perenne* in response to saline stress**

Hai-Shun Xu ^1*^, Su-Ming Guo ^1^, Lin Zhu ^2^, Jin-Cheng Xing^3*^

^1^ College of Landscape Architecture, Nanjing Forestry University, Nanjing City, Jiangsu Province, 210037, P. R. China

^2^ Design Institution of Wujin Planning and Surveying, Changzhou City, Jiangsu Province, 213100, P. R. China

^3^ Jiangsu Coastal Area Institute of Agricultural Sciences, Yancheng City, Jiangsu Province, 224000, P. R. China

^*^Corresponding to: H-S Xu (E-mail: [nj_xuhaishun@163.com](mailto:nj_xuhaishun@163.com), Tell: +86-25-85427090), J-C Xing, (E-mail: sdauxxx@163.com, Tell: +86-15961931851)

**
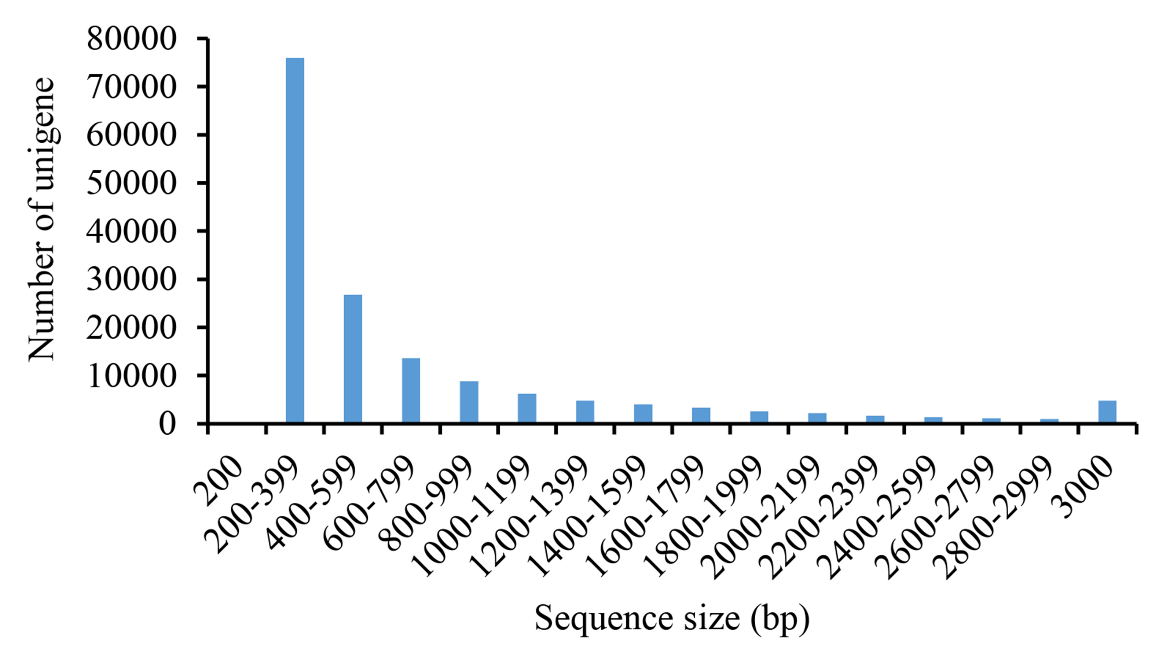
**

**Figure S1. Length distribution of unigenes**

**
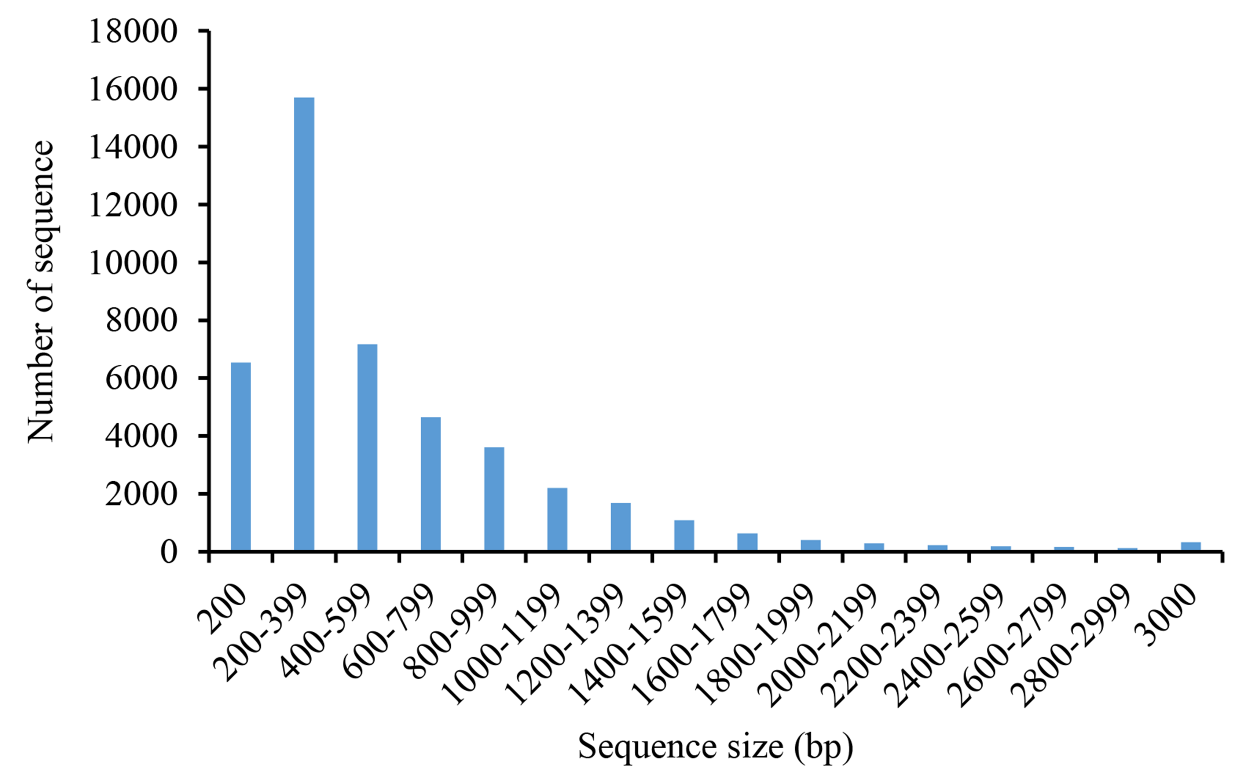
**

**Figure S2. Length distribution of CDS.**


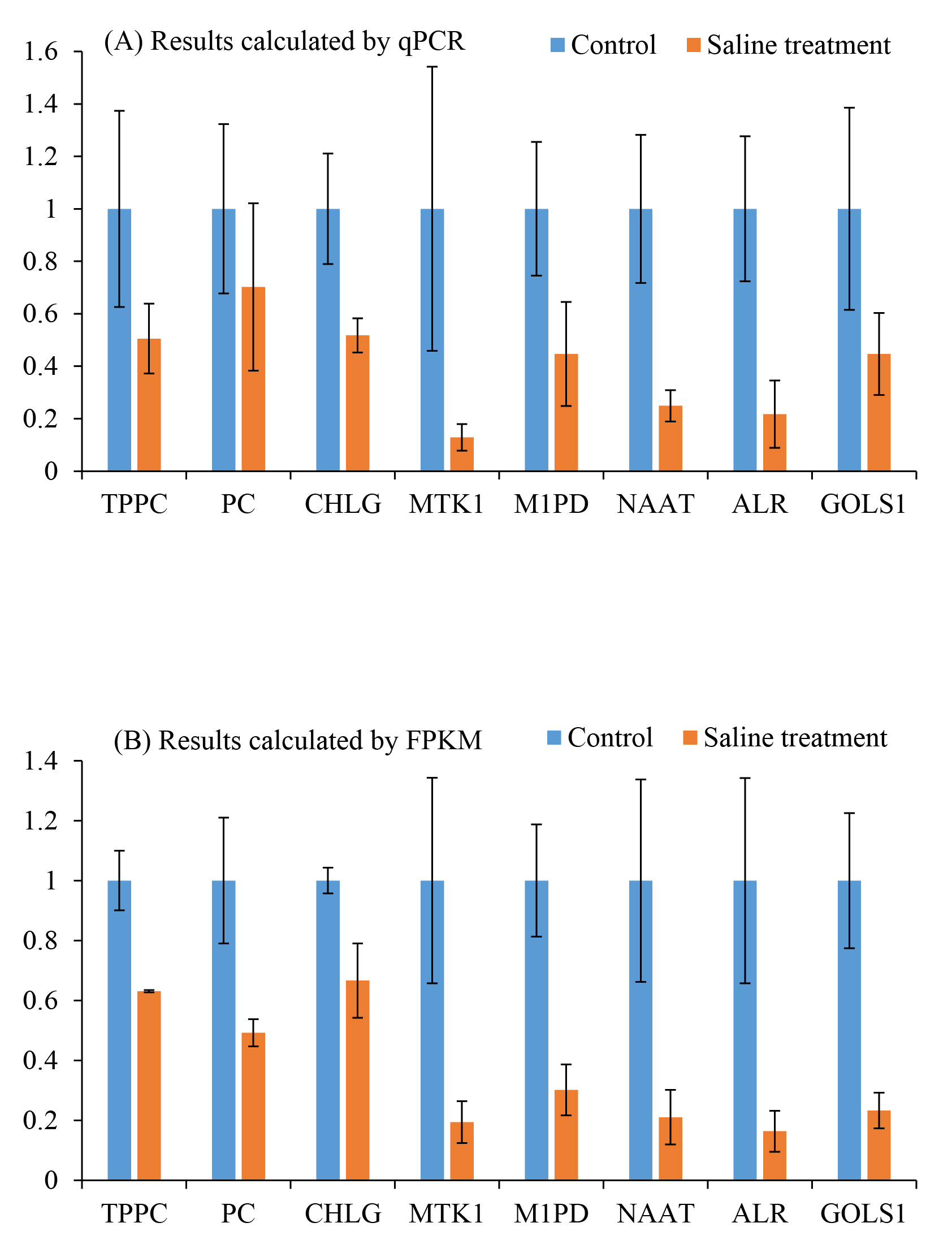


**Figure S3. Real-time qPCR validation of Illumina sequencing results (mean ± SD).**


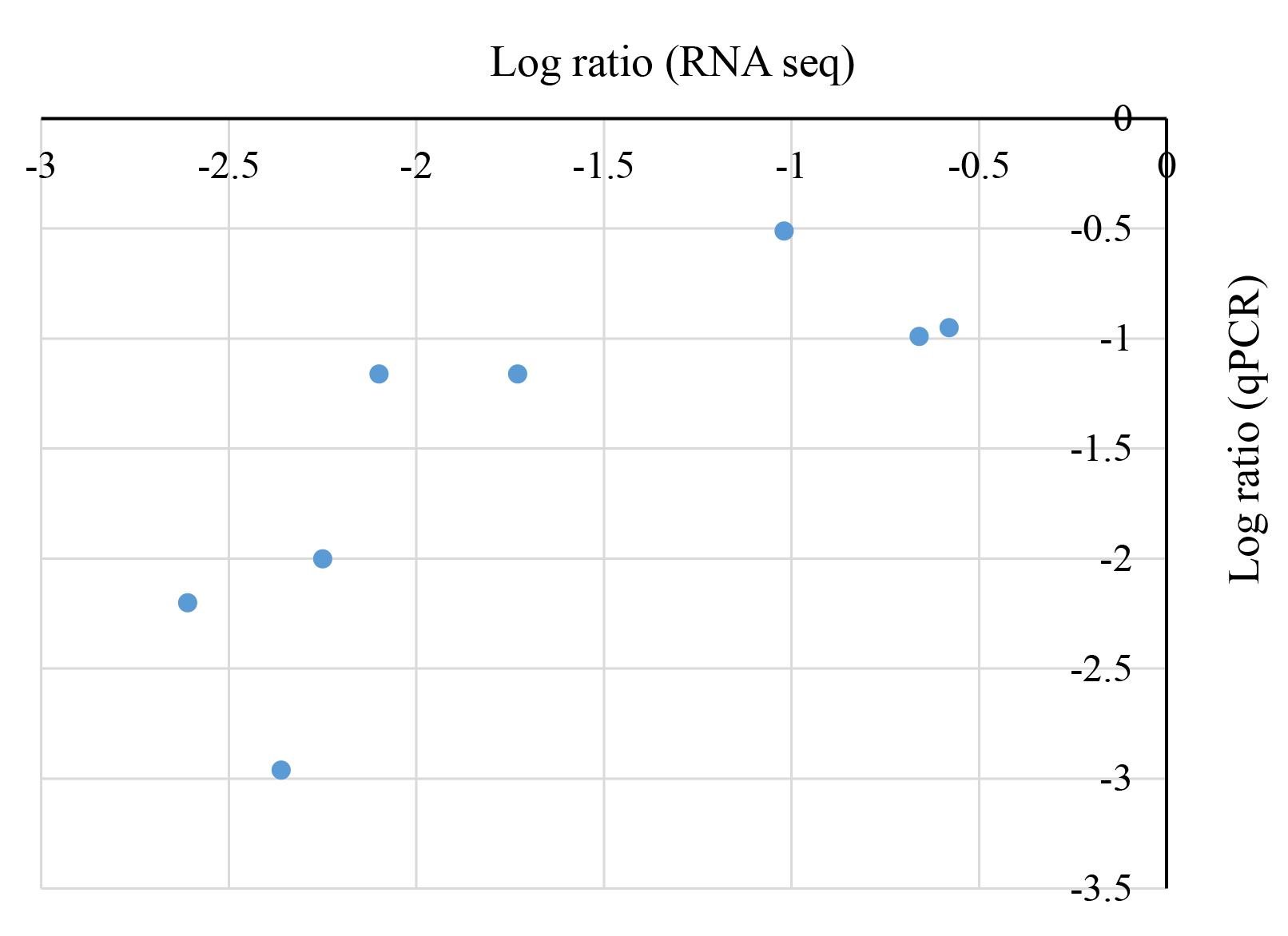


**Figure S4. Overall correlation analysis of transcriptome and qPCR**


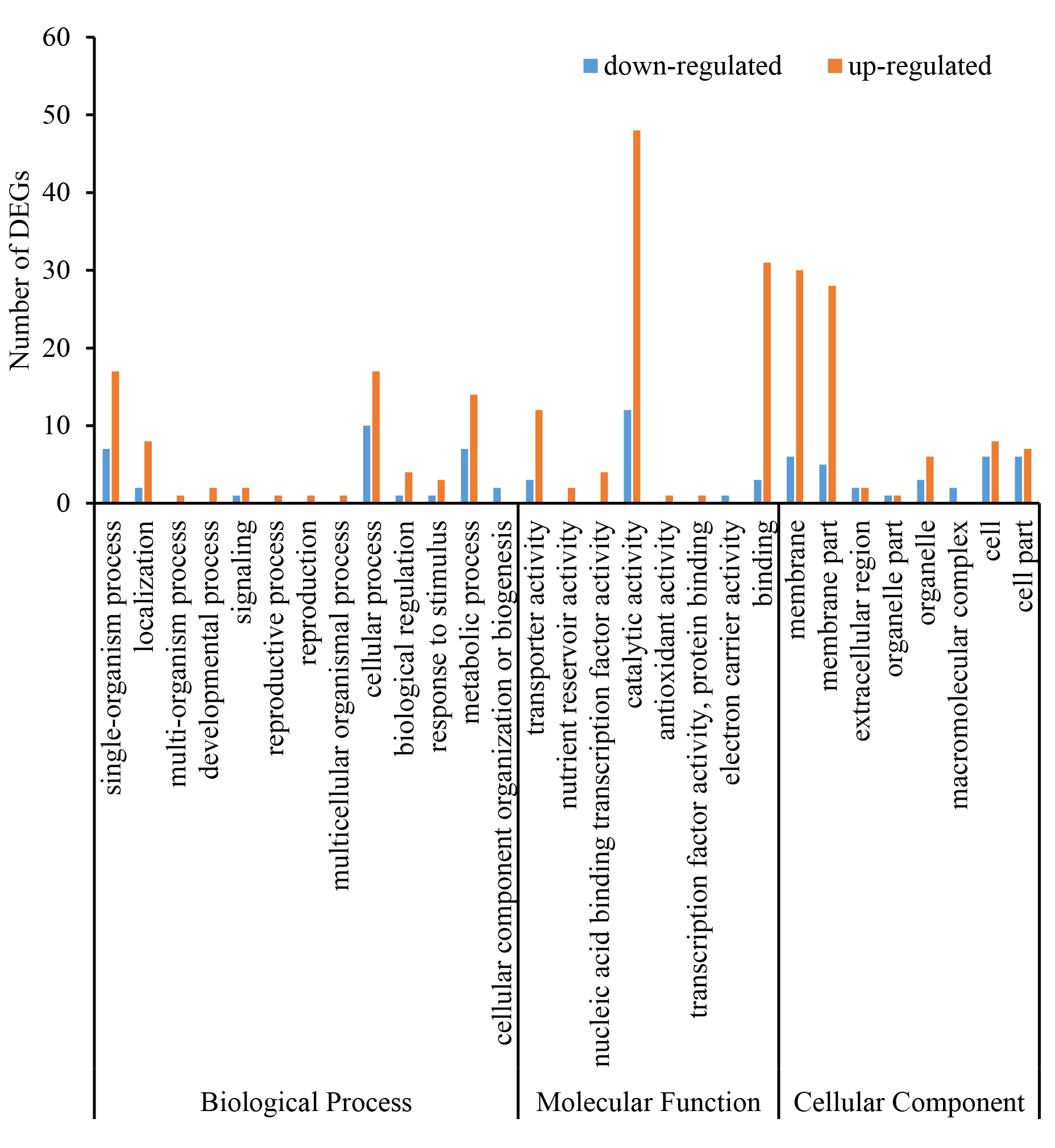


**Figure S5. GO categories of DEGs in *Lolium perenne* shoots in response to saline stress.**

**Table S1. Primers used in the present study**

| Gene description | Direction | Primer sequence (5' to 3') |
| --- | --- | --- |
| Two-pore potassium channel (TPPC) | Forward | TGCCTTGCAGTTTTTGGAGC |
|  | Reverse | TTCCAGCTTCCAGTGTGCAA |
| Potassium channel (PC) | Forward | ATGGTCAGTGTCTCGCTGTG |
|  | Reverse | TGCTTCTAGGCAGGTTCAGC |
| Chlorophyll synthase (CHLG) | Forward | TCTTGGGTTGGGTGCTTTGT |
|  | Reverse | AGCCAATGTAACTCGCACCA |
| Methylthioribose kinase 1 (MTK1) | Forward | GAGCTTTTGAGCGTTGCACA |
|  | Reverse | TCGTCCCTTCAGGATCACCT |
| Methylthioribulose-1-phosphate dehydratase (M1PD) | Forward | TGATGCTGCCGTCAAGATGT |
|  | Reverse | TACGCAATCTTTTGGCGCTG |
| Nicotianamine aminotransferase (NAAT) | Forward | CCGGGTCTTGGAAGCAGAAT |
|  | Reverse | AGCTCAATTGCTACCCACCC |
| Aldehyae reductase (ALR) | Forward | ACTGCCTCATCACCAAGAGC |
|  | Reverse | GTCATATCTCGCCGTCCCAG |
| Galactinol synthase 1 (GOLS1) | Forward | CTACTCCACATAGCAGGCGG |
|  | Reverse | TTCGGTGGCTGGTAACTGTC |
| GAPDH | Forward | AGCTCAGGAATGACCTTGCC |
|  | Reverse | ATGACCACCGTCCACTCCTA |

**Table S2. Statistics of unigenes assembled based on all samples**. N50: a weighted median statistic in which 50% of the total length is contained in unigenes greater than or equal to this value. GC (%): the percentage of G and C bases in all unigenes.

| Genes number | GC percentage | N50 | Max length | Min length | Average length | Total assembled bases |
| --- | --- | --- | --- | --- | --- | --- |
| 158,198 | 52.38 | 1,229 | 17,303 | 201 | 748 | 118,345,388 |

**Table S3. Differentially expressed genes (DEGs) in saline treatment and the control in *L. perenne* roots**

| Gene ID | Annotation | Log_2_FC | P value | Regulation |
| --- | --- | --- | --- | --- |
| Unigene0017973 | Unknown | -15.58 | 3.29E-06 | Down |
| Unigene0024652 | Unknown | 15.62 | 1.89E-06 | Up |
| Unigene0031097 | Unknown | -6.59 | 2.33E-08 | Down |
| Unigene0031502 | Unknown | 13.76 | 6.12E-11 | Up |
| Unigene0037572 | Dehydrin DHN3 | 5.39 | 4.01E-06 | Up |
| Unigene0045564 | Unknown | 13.07 | 7.55E-07 | Up |
| Unigene0047087 | Unknown | 16.34 | 1.48E-07 | Up |
| Unigene0047200 | Unknown | 12.04 | 3.33E-07 | Up |
| Unigene0064221 | Unknown | 6.53 | 3.86E-09 | Up |
| Unigene0106040 | Unknown | 15.26 | 1.88E-06 | Up |
| Unigene0110387 | Unknown | 15.27 | 2.1E-07 | Up |
| Unigene0111192 | Unknown | -11.71 | 1.79E-07 | Down |
| Unigene0114777 | Unknown | 16.07 | 2E-08 | Up |
| Unigene0142238 | Probable F-box protein At5g04010 | -13.33 | 3.06E-07 | Down |

**Table S4. FPKM values of selected genes in saline treatment and the control in *L. perenne* shoots.** Data represent mean ± standard deviation of FPKM values (n = 3). * significantly different from the control.

| Gene name | Control | Saline treatment |
| --- | --- | --- |
| Antioxidant | | |
| Superoxide dismutase [Fe] | 30.02 ± 8.47 | 26.12 ± 16.34 |
| Superoxide dismutase [Mn] | 30.83 ± 7.73 | 48.67 ± 29.8 |
| Superoxide dismutase [Cu-Zn] | 68.75 ± 5.41 | 79.72 ± 13.74 |
| Peroxidase | 10712.27 ± 1172.35 | 13057.99 ± 954.24 |
| Chlorophyll synthesis | | |
| Glutamyl-tRNA reductase | 116.26 ± 8.89 | 120.65 ± 4.14 |
| Protoporphyrinogen oxidase | 6.24 ± 1.24 | 8.1 ± 1.92 |
| Magnesium-chelatase | 8.93 ± 2.33 | 10.64 ± 3.24 |
| Chlorophyll synthase | 15.31 ± 3.78 | 7.61 ± 1.42* |
| glutathione synthetase | 30.15 ± 3.26 | 23.34 ± 1.17 |
| Iron transportation | | |
| Sodium/hydrogen exchanger | 126.7 ± 29.24 | 126.96 ± 10.07 |
| Cation/H(+) antiporter | 104.39 ± 22.32 | 163.59 ± 40.72 |
| K(+) efflux antiporter | 73.79 ± 6.8 | 92.07 ± 7.69 |
| Sodium/proton antiporter | 2.14 ± 0.45 | 1.86 ± 0.08 |
| V-type proton ATPase | 1824.33 ± 148.83 | 1429.95 ± 88.97 |
| Cyclic nucleotide-gated ion channel | 98.92 ± 10.73 | 96.81 ± 3.8 |
| Potassium channel | 73.12 ± 14.62 | 41.43 ± 1.74* |
| Two-pore potassium channel | 217.23 ± 20.49 | 139.46 ± 6.86* |
| Chloride channel protein | 90.89 ± 3.61 | 94.75 ± 7.96 |
| Cadmium/zinc-transporting ATPase | 38.42 ± 2.82 | 45.59 ± 3.33 |
| Calcium-transporting ATPase | 2933.49 ± 405.66 | 3520.25 ± 517.75 |
| Copper-transporting ATPase | 308.37 ± 22.83 | 238.2 ± 29.42 |
| Phospholipid-transporting ATPase | 184.13 ± 12.23 | 188.12 ± 13.5 |
| Plasma membrane ATPase | 472.17 ± 17.38 | 544.1 ± 50.34 |
| Galactose metabolism | | |
| aldehyae reductase | 709.5 ± 242.73 | 123.73 ± 47.56* |
| 6-phosphofructokinase 1 | 13.45 ± 4.22 | 67.33 ± 15.25* |
| galactinol synthase 1 | 30.48 ± 6.86 | 7.11 ± 1.81* |
| alpha-galactosidase | 0.54 ± 0.21 | 5.22 ± 2.46* |
| beta-fructofuranosidase 1 | 10.29 ± 1.91 | 41.77 ± 4.36* |
| Fructose and mannose metabolism | | |
| aldehyae reductase | 709.5 ± 242.73 | 123.73 ± 47.56* |
| WRKY transcription factor | 26.5 ± 2.06 | 170.81 ± 62.43* |
| fructose-bisphosphate aldolase | 4.67 ± 3.22 | 80.42 ± 19.28* |
| 6-phosphofructokinase 1 | 13.45 ± 4.22 | 67.33 ± 15.25* |
| Cysteine and methionine metabolism | | |
| Spermine synthase | 2.69 ± 0.24 | 8.54 ± 0.95* |
| Methylthioribose kinase 1 | 96.31 ± 33.01 | 18.72 ± 6.74* |
| Methylthioribose-1-phosphate isomerase | 426.02 ± 98.82 | 70.2 ± 27.03* |
| methylthioribulose-1-phosphate dehydratase | 114.39 ± 21.39 | 34.5 ± 9.71* |
| 1,2-dihydroxy-3-keto-5-methylthiopentene dioxygenase 2 | 174.78 ± 50.25 | 57.39 ± 4.5* |
| Nicotianamine aminotransferase | 5100.46 ± 1666.5 | 907.47 ± 392.97* |
| Methionine gamma-lyase | 3.93 ± 1.45 | 28.9 ± 12.75* |
| aminocyclopropanecarboxylate oxidase | 0 ± 0 | 9.92 ± 5.67* |
| Phenylalanine, tyrosine and tryptophan biosynthesis | | |
| Indole-3-glycerol phosphate synthase | 4.47 ± 0.57 | 25.05 ± 9.3* |
| Nicotianamine aminotransferase | 5100.46 ± 1666.5 | 907.47 ± 392.97* |
| Tyrosine metabolism |  |  |
| Glutathione S-transferase | 12.83 ± 1.42 | 3.47 ± 0.22* |
| Phenylalanine metabolism | | |
| Nicotianamine aminotransferase | 5100.46 ± 1666.5 | 907.47 ± 392.97* |
| Trans-cinnamate 4-monooxygenase | 38.18 ± 3.63 | 227.88 ± 8.89* |
